# Gene–Folic Acid Interactions and Risk of Conotruncal Heart Defects: Results from the National Birth Defects Prevention Study

**DOI:** 10.3390/genes14010180

**Published:** 2023-01-09

**Authors:** Daniel M. Webber, Ming Li, Stewart L. MacLeod, Xinyu Tang, Joseph W. Levy, Mohammad A. Karim, Stephen W. Erickson, Charlotte A. Hobbs, The National Birth Defects Prevention Study

**Affiliations:** 1 Department of Pathology and Immunology, Washington University School of Medicine, St. Louis, MO 63110, USA; 2 Department of Epidemiology and Biostatistics, Indiana University at Bloomington, Bloomington, IN 47405, USA; 3 Division of Birth Defects Research, Department of Pediatrics, University of Arkansas for Medical Sciences, Little Rock, AR 72205, USA; 4 Biostatistics Program, Department of Pediatrics, University of Arkansas for Medical Sciences, Little Rock, AR 72205, USA; 5 Department of Obstetrics and Gynecology, Wayne State University, Detroit, MI 48202, USA; 6 Department of Child Health, College of Medicine, University of Arizona, Phoenix, AZ 85004, USA; 7 Department of Neurology, Sections on Neurodevelopmental Disorders, Barrow Neurological Institute at Phoenix Children’s Hospital, Phoenix, AZ 85016, USA; 8 Center for Genomics in Public Health and Medicine, RTI International, Research Triangle Park, NC 27709, USA; 9 Rady Children’s Institute for Genomic Medicine, Rady Children’s Hospital, San Diego, CA 92123, USA

**Keywords:** folate, congenital, heart, NBDPS, *MTHFS*, gene environment

## Abstract

Conotruncal heart defects (CTDs) are heart malformations that affect the cardiac outflow tract and typically cause significant morbidity and mortality. Evidence from epidemiological studies suggests that maternal folate intake is associated with a reduced risk of heart defects, including CTD. However, it is unclear if folate-related gene variants and maternal folate intake have an interactive effect on the risk of CTDs. In this study, we performed targeted sequencing of folate-related genes on DNA from 436 case families with CTDs who are enrolled in the National Birth Defects Prevention Study and then tested for common and rare variants associated with CTD. We identified risk alleles in maternal *MTHFS* (OR_meta_ = 1.34; 95% CI 1.07 to 1.67), maternal *NOS2* (OR_meta_ = 1.34; 95% CI 1.05 to 1.72), fetal *MTHFS* (OR_meta_ = 1.35; 95% CI 1.09 to 1.66), and fetal *TCN2* (OR_meta_ = 1.38; 95% CI 1.12 to 1.70) that are associated with an increased risk of CTD among cases without folic acid supplementation. We detected putative de novo mutations in genes from the folate, homocysteine, and transsulfuration pathways and identified a significant association between rare variants in *MGST1* and CTD risk. Results suggest that periconceptional folic acid supplementation is associated with decreased risk of CTD among individuals with susceptible genotypes.

## 1. Introduction

Congenital heart defects are the most-common congenital abnormality [1]. Both genetic and environmental factors have been implicated in the occurrence of congenital heart defects [2]. Evidence suggests that dietary folic acid fortification and use of a folate-containing prenatal multivitamin are associated with a decreased risk of heart defects, including conotruncal heart defects (CTDs) [3,4,5]. Research from our laboratory and others has shown that maternal and fetal genetic variants in the folate, homocysteine, and transsulfuration pathways are associated with an increased risk of heart defects, specifically CTDs [6,7]. However, it is unclear if the effects of variants in these folate-related pathways on CTD risk are modified by maternal folate intake.

CTDs are life-threatening heart malformations that affect cardiac outflow tracts and great arteries. Our laboratory has previously reported single-nucleotide polymorphisms (SNPs) in the folate, homocysteine, and transsulfuration pathways that were independently associated with CTDs, and we identified SNPs that were only associated with CTD risk when folic acid supplementation was considered. These results highlighted the need for further study and replication. Therefore, this study identifies and characterizes variants associated with CTDs within candidate genes of the folate, homocysteine, and transsulfuration pathways. Specifically, we sought to (1) replicate significant gene–folic acid interactions among CTD susceptibility loci from our initial study, and (2) identify rare and de novo variants in CTD-susceptibility loci.

Here, we present data showing that periconceptional folic acid supplementation is consistently associated with a lower risk of CTD among families with risk alleles in *MTHFS*, *NOS2*, or *TCN2*. We also report putative de novo mutations in folate-related genes that are significantly enriched among cases, and we identify rare variants within *MGST1* that are associated with risk of CTD.

## 2. Materials and Methods

### 2.1. Compliance with Ethical Standards

This study was approved by the Institutional Review Boards of the Centers for Disease Control and Prevention (CDC), the University of Arkansas for Medical Sciences, and the other study sites in the National Birth Defect Prevention Study (NBDPS). Informed consent was obtained for all study participants. The collection of data and biologic samples was funded by the Centers for Disease Control and Prevention’s National Center on Birth Defects and Developmental Disabilities and was coordinated by the National Birth Defects Prevention Study. The authors have no conflicts of interest, either financial or non-financial, in the subject matter discussed in this manuscript.

### 2.2. Study Population

We refer here to our initial work of identifying CTD risk alleles as the discovery phase and refer to the targeted resequencing of CTD-susceptibility loci as the replication phase. Participants in the discovery phase (*n* = 4648) were initially genotyped using a custom DNA microarray that included 1536 SNPs that were represented across 60 genes within the folate, homocysteine, and transsulfuration pathways. We sequenced buccal-cell DNA from 536 of the 4648 participants from the discovery phase and 740 additional participants in the replication phase. Both the discovery and replication phases used a family-based, case-control hybrid design that included case and control families from the National Birth Defect Prevention Study (NBDPS). Details of the two study phases are provided in Table 1.

The NBDPS represents one of the largest population-based studies of non-syndromic birth defects ever conducted, covering an annual birth population of 482,000, or 10% of US births [8,9,10]. Cases for this study were identified across 10 NBDPS sites using state birth defects surveillance systems. Cases included live births, stillbirths, and elective terminations with one or more of the following conotruncal heart defects: truncus arteriosus, interrupted aortic arch type B, transposition of great arteries, double outlet right ventricle, conoventricular septal heart defects, tetralogy of Fallot, and pulmonary atresia with ventricular septal defect. Controls were randomly selected at each NBDPS site from birth hospitals or birth certificates.

We used DNA samples from case and control families enrolled in the NBDPS from 1998 to 2011 to test the hypothesis that genetic variants in folate-related pathways are risk factors for CTD. The majority of families included in this study were complete mother–father–infant trios; however, mother–infant dyads, father–infant dyads, mother–father dyads, mother-only, father-only, and infant-only samples were genotyped as well (Supplementary Table S1). Mothers participating in the NBDPS were asked by phone interview about vitamin use during and prior to pregnancy, including the brand, frequency, duration, and timeframe of vitamin use. Women with periconceptional folic acid supplementation were identified as those reporting use of folic-acid-containing vitamins or supplements during the month before pregnancy or the first three months of gestation.

Sample inclusion criteria from the replication phase mirrored those of the discovery phase, except the replication phase was limited to samples with at least 50 ng of available DNA and a call rate of at least 90% if previously genotyped as part of the discovery phase.

### 2.3. Selection of Targeted Regions and Sequencing

In the discovery phase, we genotyped 1536 SNPs within 60 candidate genes in the homocysteine, folate, and transsulfuration pathways using Illumina’s Golden Gate^TM^ custom platform (San Diego, CA, USA). SNPs were selected for genotyping in the discovery phase with the aim of providing a maximally informative set of haplotype-tagging SNPs that represented common variants (MAF > 0.1) across the 60 targeted genes [6]. In the replication phase, we sequenced exons, introns, and 1 kb upstream and downstream regions of 13 folate-related genes that were selected based on the rank order of gene variants from the discovery phase (prioritizing low Bayesian False Discovery Probability, BFDP) and the number of CTD-associated variants (BFDP < 0.8) within each gene. The 13 genes that were sequenced in the replication phase include (i) glutamate–cysteine ligase catalytic subunit (*GCLC*); (ii) serine hydroxymethyltransferase 1 (*SHMT1*); (iii) nitric oxide synthase 2 (*NOS2*); (iv) *5-*methyltetrahydrofolate-homocysteine methyltransferase reductase *(MTRR*); (v) 5,10-methenyltetrahydrofolate synthetase *(MTHFS*); (vi) glutaredoxin (*GLRX*); (vii) DNA methyltransferase 3 α (*DNMT3A*); (viii) superoxide dismutase 2, mitochondrial (*SOD2*); (ix) microsomal glutathione s-transferase 1 (*MGST1*); (x) glutathione peroxidase 4 (*GPX4*); (xi) transcobalamin 2 (*TCN2*); (xii) methylenetetrahydrofolate dehydrogenase (NADP + dependent) 2, methenyltetrahydrofolate cyclohydrolase (*MTHFD2*); (xiii) and the enolase superfamily member 1 (*ENOSF1*)/thymidylate synthetase (*TYMS*) gene loci (*ENOSF1/TYMS*).

### 2.4. Sample Preparation

DNA was collected from the mother, father, and infant and was processed using well-established NBDPS and local Arkansas protocols [9]. Illumina Nextera hybridization enrichment was used to capture 13 candidate exons, introns, and 1 kb upstream and downstream regions from 50 ng per sample of genomic DNA. Target libraries were pooled at 48× and sequenced on an Illumina HiScan-SQ and NextSeq500.

### 2.5. Data Processing and Genotype Calling

Sequencing data were processed using Genome Analysis Toolkit (GATK) Best Practices for Germline SNPs and Indels in Whole Genomes and Exomes [11]. The basic steps are outlined here. Raw sequencing data were demultiplexed and converted to FASTQ format by CASAVA v1.8.2 and Illumina BaseSpace. Sequences were mapped to the reference genome (GRCh37/hg19) with the Burrows–Wheeler Aligner (BWA) and MEM algorithm [12]. Duplicate sequence reads were marked with Picard Tools. Base quality scores were recalibrated with GATK v3.3. GATK Haplotype Caller was used to generate per-sample Genomic VCF files, which were genotyped jointly to produce a multi-sample VCF. Resulting single-nucleotide variants were filtered out if any of the following criteria were met: quality by depth (QD) < 2.0, Fisher strand bias (FS) > 60.0, RMS mapping quality (MQ) < 40.0, haplotype score > 13.0, mapping quality rank sum < −12.5, read position rank sum test < −8.0. Indels were filtered out based on the following criteria: QD < 2.0, FS > 200.0, Read Position Rank Sum < −20.0. Filtered variants were phased among families using GATK PhaseByTransmission [13]. SnpEff v4.0 was used to annotate genomic variants with conservation and predictor scores from the dbNSFP v2.0 database [14].

### 2.6. Quality Control

Thirty-one samples from the present study were also genotyped with an Illumina HumanOmni 5M BeadChip as part of another project. Genotype calls were compared at 196 overlapping sites. The concordance rate across platforms was 99.0% before imputations, indicating high accuracy in variant calls. All samples were screened for Mendelian inconsistencies using PLINK [15]. Statistical analyses were independently replicated by a second statistician per NBDPS protocol. Sequencing methods were validated using a well-characterized sample from the 1000 Genomes project [16] as part of an NBDPS external quality assurance procedure.

In our replication phase, all families and SNPs showed Mendelian error rates of less than 5%. All genotypes with Mendelian inconsistency were set to missing. To ensure the genotyping quality, we further removed SNPs with a call rate of less than 95% and participants with a call rate of less than 70%.

### 2.7. Imputation

The targeted sequencing provided genotypes for 9402 variants in the replication phase. However, only 74 of those variants were genotyped in the discovery phase. To maximize the overlap of variants between the two phases, we conducted genotype imputation for each phase by using 1000 Genomes Project Phase 3 Genome Build b37 as reference panels. Software IMPUTE version 2 was used for the imputation with a “multi-population reference panels” option, which chooses a “custom” reference panel for each individual based on all available reference haplotypes [17,18]. The advantages of such a strategy have been discussed elsewhere [19]. After the imputation and quality control, the discovery phase and the replication phase included 3505 variants and 9588 variants, respectively. A total of 2945 variants overlapped between the two phases. We further compared the genotype concordance between two phases based on these 2945 variants from 536 overlapping samples. The genotype concordance across platforms was estimated to be 97.9%, indicating a robust imputation result.

### 2.8. Gene–Folic Acid Interaction Analysis

There is strong evidence that periconceptional folic acid intake decreases the risk of conotruncal heart defects [20,21,22,23,24,25,26,27]. Folate-related genes, such as *MTHRF,* are known to influence the risk of heart defects via interactions with maternal folic acid intake [28]. We, therefore, hypothesized that genetic risk factors in CTD might be modified by periconceptional folic acid supplementation. We used an extended log-linear model, first proposed by Weinberg and Umbach, to investigate gene–folic acid interactions for each of the common variants [29]. This model simultaneously estimates the effects of maternal and fetal genotypes for a given SNP on disease risk [29]. It is also worthwhile to note that this model was initially proposed for analysis of case-parental trios and does not require samples from the control families [30]. Therefore, the estimated genetic relative risk does not rely on the contrast between case and control families. Additional details of the analysis are provided in the Supplementary File S1. We and others have previously used such modeling strategies for gene-by-environment analysis in studies with trio designs [6,7,31]. For the identified genetic variants, we report the relative genetic risk among 154 folate-supplemented families and 99 folate-unsupplemented families from the replication study phase.

We applied the log-linear model to the discovery phase and replication phase separately and further combined the results from both phases by fixed-effect meta-analysis with inverse variance weighting. The significance level of the interaction effect is evaluated by the Bayesian false-discovery probability (BFDP) for the meta-analysis results. When the associations between genetic variants and disease risk were consistent between discovery and replication phases, the BFDP for meta-analysis was expected to be smaller than those estimates from either the discovery or replication phases. Additional details of meta-analysis and BFDP derivatives are provided in the Supplementary File S1.

The BFDP assesses the noteworthiness of an association by balancing the costs of false discovery and non-discovery [32] and has been widely used in genetic association studies [33,34,35,36,37]. We used a widely accepted BFDP threshold of less than 0.8 for reporting noteworthy associations. A BFDP threshold of 0.8 implies that not reporting a noteworthy association (i.e., false non-discovery) is four-times as costly as reporting a non-noteworthy association (i.e., false discovery).

The statistical analysis was conducted in R 3.1.3 (R Development Core Team, Vienna, Austria) using the LEM package for log-linear modeling [38] and the METAL package for meta-analysis [39,40].

### 2.9. Detection and Testing of De Novo Variants

Phased genotyping calls from case and control trios were refined using GATK v3.3, based on the GATK genotype refinement workflow [11]. GATK VariantAnnotator was then used to annotate potential de novo mutations present within the set of refined, high-quality genotypes. This process resulted in 29 candidate de novo mutations among cases and 0 candidate mutations among controls. Further filtering of candidates based on quality and read depth yielded 12 likely de novo mutations among the cases. The observed frequency of likely de novo mutations among cases was compared to the predicted frequency within our targeted regions, based on the genome-wide human mutation rate map from Genome of the Netherlands Consortium [41,42].

### 2.10. Region-Based Analysis for Rare Variants

We conducted region-based association tests for rare variants (i.e., MAF < 5%) within each of the 13 targeted regions using the full replication dataset. The analysis was conducted by using two approaches: the rare-variant extensions of the transmission disequilibrium test (RV-TDT) and the family-based sequence kernel association test (FB-SKAT) [43,44]. Because these methods focus on the transmission of alleles from parents to offspring, they are tolerant of population substructure and admixture. It was shown from previous studies that the two approaches may have their unique advantages under different disease etiology. The RV-TDT may have improved power over FB-SKAT when a relatively high proportion of variants is causal with the same direction of effect size (i.e., deleterious or protective), while FB-SKAT may have improved power when a relatively low proportion of variants is causal or they have bi-directional effects [45].

## 3. Results

### 3.1. Population

This study included 1276 participants from 476 families (436 case families and 40 control families) whose buccal-cell DNA underwent targeted sequencing, alignment of sequence reads to the human genome, variant calling, variant imputations, and quality control (see Materials and Methods for details on sample processing and Table 2 for demographic information). In total, 536 of these 1276 participants were previously genotyped in the discovery phase using a custom DNA microarray that covered 1536 SNPs within 60 genes in the folate, homocysteine, and transsulfuration pathways [6]. The remaining 740 of 1276 participants were not previously analyzed and were thus included in the replication phase. We utilized a hybrid log-linear model to test for interactions between prenatal folate supplementation and the genotype of 2945 SNPs that were represented across the 4684 participants in the discovery phase and 740 independent samples sequenced in the replication phase. Demographics for the discovery and replication sample sets are displayed in Supplementary Table S2.

Secondary aims for this study included (i) testing for an excess of de novo mutations within candidate genes and (ii) conducting region-based association tests for rare variants. These subsequent tests utilized the full set of 1276 participants from 476 families that were sequenced in this study and passed quality control, including 360 complete trios, 55 mother–infant dyads, 12 father–infant dyads, 13 mother–father dyads, 10 mother singlets, 2 father singlets, and 24 infant singlets (Supplementary Table S1). The majority of case and control mothers were non-Hispanic white, had some college education, earned less than USD 30,000, reported folic acid supplementation, and did not smoke or consume alcohol. Tetralogy of Fallot and Transposition of great arteries were the two most-common types of conotruncal heart defects included in the study, representing 175/422 (41.5%) and 138/422 (32.7%) of cases, respectively (see Supplementary Table S3 for phenotypic distribution).

### 3.2. Gene–Folic Acid Interaction

The fraction of case mothers receiving periconceptional folate supplementation was not significantly different between specific types of conotruncal heart defects (*p*-value = 0.59, Fisher’s exact test), as is shown in Supplementary Table S3. We identified 54 significant interactions (*p*_meta_ < 0.05, BFDP_meta_ < 0.8) between SNPs and folate supplementation status (Supplementary Table S4), such that the minor allele of either the mother or the infant increased the risk of CTD among infants without folic acid supplementation. Thirty-three of these SNPs had significant infant gene-by-folate interactions. These 33 SNPs were part of two linkage disequilibrium (LD) blocks: one in *MTHFS* and one in *TCN2* (see Supplementary Figure S1A,B for LD plots of SNPs with significant infant gene-by-folate interactions). The remaining 21 SNPs had significant maternal gene-by-folate interactions and were part of an LD block in *NOS2* and one in *MTHFS* (see Supplementary Figure S1C,D for LD plots of SNPs with significant maternal gene-by-folate interactions). Supplementary Figure S2 depicts the workflow for gene–folic acid interaction analysis as it relates to SNP discovery and validation in the two phases of this study.

Table 3 displays the lead SNPs with the strongest interaction effect (lowest metanalysis *p*-value) for each LD block. The SNP, rs12438477, in *MTHFS* was notable for having the strongest fetal gene-by-folate interaction effect (*p*_meta_ = 4.02 × 10^−5^ and BFDP_meta_ = 0.12). Among mothers reporting no periconceptional folic acid supplementation, the fetal minor allele (A) of rs12438477 was estimated to increase the risk of CTDs in the meta-analysis by approximately 1.41-fold per copy, 95% CI [1.11, 1.79]. Among mothers reporting periconceptional folic acid supplementation, the fetal minor allele appears to be protective for CTD (meta-analysis relative risk = 0.74, 95% CI (0.61, 0.92)). The direction and magnitude of effects were consistent across the discovery and replication studies.

### 3.3. De Novo Mutations

Although de novo mutations do not contribute directly to association signals, these mutations can help identify the precise location of disease-associated variants. We identified 12 de novo mutations based on the targeted sequencing data of 365 case-parental trios. Table 4 shows likely de novo mutations identified in the replication phase. All but two of the mutations affected regulatory regions of introns, such as transcription factor binding sites (TFBSs) and DNase footprints. Mutations at chr2:25,460,216 and chr15:8,012,657 did not appear to affect regulatory DNA; instead, these variants were predicted to affect the strength of cryptic splice sites. The likely de novo mutation, rs568740763, was notable in that it affected the *ENOSF1* 3′ un-translated (UTR) and appeared to provide a new Poly-A-signal for the *ENOSF1* protein. All 12 of these putative de novo mutations were at low allele frequency in the general population (AF < 1%) or were absent from the Genome Aggregation Database v3.1.2, which contains 76,156 whole genomes [46].

The normal mutation rate for the selected sequenced regions was estimated to be *p*_0_ = 3.90408 × 10^−8^, based on the genome-wide human mutation rate [41,42]. We tested for enrichment of de novo mutations within our case samples by using an exact test of binomial distribution. Under the null hypothesis that the mutation rate among our samples was no different from the normal rate (i.e., $H_{0}:p=p_{0}$), the probability of observing at least 12 de novo mutations among 365 trios is 1.22 × 10^−67^, as shown below:

$$\overset{k=365}{\underset{k=12}{\sum }}\left(\begin{array}{c} 365\\ k \end{array}\right)p_{0}{}^{k}{\left(1-p_{0}\right)}^{365-k}=1.22\times 10^{-67}$$

This result indicates that the actual mutation rate among infant cases is higher than expected, assuming that the baseline rate of de novo mutations in the case population is similar to that of healthy individuals represented by the genome-wide human mutation rate map from Genome of the Netherlands Consortium [41,42].

### 3.4. Region-Based Analysis for Rare Variants

We used the FB-SKAT and RV-TDT methods to test for unexpectedly high transmission of genetic variants from parents to offspring. We applied these methods to each of the 13 genes and restricted the analysis to variants with a low allele frequency (MAF < 5%). Whereas the analysis described above (Gene–Folic Acid Interaction section) is well-suited to identifying common, disease-associated variants, these two methods are well-suited to identifying disease-associated regions/genes. These two region-based methods are complementary and their performance and results depend on disease mechanisms as discussed in the Materials and Methods section. 

The results of FB-SKAT and the RV-TDT analyses are shown in Table 5. With a Bonferroni-corrected threshold for 13 regions (i.e., 0.05/13 = 0.0038), only *MGST1* showed statistical significance when analyzed by FB-SKAT. It is likely that this result was discordant with RV-TDT because of how these methods account for differences in the effect size and direction of variants within a region. *GLRX* and *GPX4* displayed nominal statistical significance (*p* < 0.05) that did not reach the Bonferroni-corrected threshold.

## 4. Discussion

### 4.1. Gene–Folic Acid Interaction

*MTHFS* polymorphisms. Testing for gene–folic acid interactions identified one maternal (rs7163338) and one fetal SNP (rs12438477) in the folate pathway gene *MTHFS* that were associated with CTD among infants with low folic acid supplementation (*p*_meta_ < 0.05, BFDP_meta_ < 0.8). MTHFS maintains the supply of the methyl donor 5,10-methenyltetrahydrofolate (5,10-methenyl-THF), which is a critical cofactor in the de novo synthesis of nucleotides thymidine, adenine, and guanine [47,48,49]. Of note, the lead *MTHFS* SNP in infants, rs12438477, was previously shown to increase the risk of congenital heart defects by 6.76-fold (95% CI [2.56,17.89]) among infants with the AA genotype and SSRI exposure [50].

*TCN2* polymorphisms. Our meta-analysis identified fetal polymorphisms in *TCN2* that were associated with an increased risk of a CTD-affected pregnancy among women with no periconceptional folic acid supplementation. The *TCN2* gene encodes transcobalamin 2, which mediates transport of B12 to peripheral tissues and enables cellular B12 uptake [51]. Both B12 and circulating folate (5-methyltetrahydrofolate) are necessary for recycling homocysteine into methionine [52]. As a result, homocysteine increases when these two compounds are deficient, and it decreases when folic acid supplementation is initiated [53]. This likely explains why polymorphisms that affect the B12 transport protein *TCN2* have been associated with higher circulating homocysteine levels in the meta-analysis [54]. Of particular importance, our lead SNP within the *TCN2* locus, rs2301957, is associated with elevated blood homocysteine levels in prior research [55]. Elevated homocysteine is a risk factor for congenital heart defects, both in humans and in model systems. The risk of congenital heart defects can be over 4-fold higher among women with elevated homocysteine levels [56], whereas studies using the avian embryo model have demonstrated that homocysteine can induce CTDs in a manner that is dependent upon dose and time of exposure [57,58,59].

Our data suggest that folic-acid-containing vitamins mitigate the risk of CTD among infants with *TCN2* polymorphisms. Folate deficiency and *TCN2* polymorphisms are both associated with increased homocysteine; thus, hyperhomocysteinemia represents a potential mechanism for congenital heart defects. However, homocysteine was not measured in this study and further research is needed to fully resolve this question.

*NOS2* polymorphisms. *NOS2* (inducible NOS) is an isoform of nitric oxide synthase (NOS) that produces nitric oxide in response to various environmental stimuli. Nitric oxide synthase is expressed and found in high abundance in heart tissue [60,61] and has multiple cardiovascular effects, including vasodilation, regulation of vessel permeability, anti-coagulation, angiogenesis, and formation of cardiac cushions [62,63,64,65].

Our results demonstrated that mothers with no folic acid supplementation and minor alleles at the *NOS2* risk loci have an increased risk of having CTD-affected offspring. The absence of folic acid supplementation predisposes these individuals to hyperhomocysteinemia [53]. Hyperhomocysteinemia is known to inhibit angiogenesis [66,67] and increase the risk of heart malformations [56,68]. Prior studies have shown that the inhibitory effects of homocysteine on angiogenesis are dependent upon *NOS2* expression [66,67,69,70]. Thus, low folic acid intake and resulting hyperhomocysteinemia may interact with *NOS2* polymorphisms to influence the risk of CTD.

*NOS2* also plays a major role in placental blood flow [71], response to infection [72], and development of diabetic embryopathy [73]. *NOS2* expression is dramatically increased in diabetic mice, and evidence suggests that excess *NOS2* contributes to diabetes-induced heart defects by increasing nitrosative stress and cell apoptosis [73,74,75]. It is unclear what effect *NOS2* risk alleles (Table 3 and Supplementary Table S4) might have on gene expression or activity within the developing heart. However, there are examples from the literature of *NOS2* gene variants within and near our risk loci, which are associated with increased NO production (e.g., rs4795067, rs2297518) [76,77].

This study replicated a maternal *NOS2* gene–folic acid interaction from the discovery phase. *NOS2* SNPs have not been well studied in the context of CTD. However, *NOS2* associations have been reported for various other conditions, including a GWAS association between rs4795067 and psoriasis [78]. This particular SNP (rs4795067) was identified as a CTD-risk allele in our study and has also been associated with neural tube defects in a large candidate gene study [79].

### 4.2. De Novo Mutations

De novo mutations were over-represented among our cases compared to reference mutation rates from the Genome of the Netherlands Consortium [41,42]. No de novo mutations were detected among 40 control families. Mutations appear to be especially common within *DNMT3A*; however, this is due, in part, to the comparatively large size of this gene (111.6 kb). Nonetheless, three of the *DNMT3A* de novo mutations are within significant regulatory regions. The variants at chr2:25,532,226 and chr2:25,548,310 affect transcription factor binding sites (TFBS) within fetal heart enhancers, which represent important regulatory regions during fetal heart development [80], whereas the mutation at chr2:25,549,757 affects a region with abundant regulatory features and modest transcription in fetal heart tissue.

### 4.3. Region-Based Association Tests

*MGST1* gene variants. Region-based association tests identified three regions that were nominally associated with CTD; however, only *MGST1* remained associated with CTD (Bonferroni-corrected *p* < 0.0038) after accounting for multiple testing. *MGST1* encodes a glutathione S-transferases enzyme, which protects the endoplasmic reticulum and outer mitochondrial membranes from xenobiotics, reactive metabolites, and oxidative stress [81]. Prior work in our lab identified rs7294985 as an *MGST1* polymorphism that influences the risk of heart defects among infants with prenatal exposure to selective serotonin reuptake inhibitors (SSRIs) [50]. Chowdhury et al. [82] identified five SNPs within *MGST1* that were associated with the ratio between reduced and oxidized glutathione (GSH/GSSG) among mothers. Cardiogenesis is sensitive to oxidative stress and xenobiotics [83,84]. It is, therefore, possible that harmful variants within *MGST1* sensitize the fetal heart to xenobiotics and reactive oxygen species.

### 4.4. Overview of Gene–Environment Interactions

GWAS studies of CTDs have identified several risk loci [85,86], including risk alleles at 12q24 and 13q32. However, prior GWAS of CTDs have not identified significant associations for the candidate genes included in this study. This may be due to the high significance threshold set by GWAS studies (1 × 10^−8^) or may be a result of unmeasured gene–folic acid interactions in GWAS that have the potential to mask significant associations [87]. As the cost of sequencing decreases, one can envision a near future with routine genetic screening. Such screening will enable targeted prevention of heart defects based on gene–environmental interactions detailed in this and other studies.

The importance of gene–environment interactions is further illustrated by the results of this study. Folic acid supplementation was included as a covariate in this analysis based on evidence of a gene–folic acid interaction in the discovery phase. Inclusion of folic acid supplementation in the model unmasked relevant genetic associations and provides useful data for targeted interventions.

### 4.5. Strengths and Limitations

Limitations. This study was limited in the number of covariates in the analysis. The log-linear model used in the analysis has many advantages, including tolerance to population stratification for fetal effect [88]. However, one of its limitations is that it is quite inflexible to the inclusion of covariates. We chose to evaluate the interaction between gene variants and folic acid supplementation rather than other potential covariates because of evidence for this interaction in the discovery phase [6]. Although this approach was well-reasoned, it does not account for potential gene–environment interactions with factors, such as maternal obesity, diabetes, and medication exposure [50,75,89,90,91,92,93]. The study also has certain biases that are inherent to targeted gene analyses. For instance, it is limited to the detection of associations within the targeted genes and is not powered to detect associations at the genome-wide significance threshold of 5 × 10^−8^. Although 1276 participants were sequenced as part of the replication study, 536 individuals were excluded from the meta-analysis due to an overlap between study phases. Nonetheless, the overlap in samples improved confidence in genotype calls and increased the density of genetic data. Furthermore, the full set of 1276 participants was utilized in rare-variant testing and de novo analysis.

Strengths. A major strength of this research was that it utilized samples from the NBDPS. The NBDPS has the advantage of being a large, multi-center, population-based, case-control study with consistency across centers afforded by well-validated protocols for case ascertainment, maternal interview, sample collection, DNA extraction, case review, and phenotype classification [94]. This analysis benefited from a large sample size consisting of 4648 participants in the discovery phase and 1319 participants in the replication phase. It also benefited from a two-phase design, which generally reduces false positives and improves the detection of genetic associations [95]. The study was strengthened by the use of complementary methods, including microarray and sequencing. The use of both methods allowed us to confirm the accuracy of genotyping calls across multiple platforms and to conduct rare-variant burden testing among sequenced samples. An additional strength of the study was its trio family design, which provided a means of detecting de novo mutations and conducting family-based analyses [38]. By testing candidate genes with clear biologic relevance, this study was able to identify low-penetrant genetic variants that influence the risk of CTD.

## 5. Conclusions

Results suggest that folic acid supplementation reduces the risk of CTD among families with a maternal-risk allele in *MTHFS* (rs7163338) or *NOS2* (rs2779248) or a fetal-risk allele in *MTHFS* (rs12438477) or *TCN2* (rs2301957). We observed an excess in likely de novo mutations in folate, homocysteine, and transsulfuration genes among case families. We also found an excess of rare variants in *MGST1* among cases with CTD, suggesting that rare variants within this gene may also be linked to CTD. These results support current recommendations by the U. S. Public Health Service and CDC that women of reproductive age take a supplement containing 400–800 µg folic acid daily [96].

## Figures and Tables

**Table 1:** Comparison of discovery and replication studies.

	Discovery Phase	Discovery Phase	Replication Phase
	Microarray ^a^	Sequencing ^b^	Sequencing ^b^
Sample size, post-QC (*n*)			
Total participants	4648	536	740
Case families	616	186	251
Case parental trios	230	97	230
Control families	1645	37	6
Control parental trios	559	29	2
Genomic Variants			
Number of assayed genes	60	13	13
Number of variants genotyped	1536	9402	9402
Number of variants imputed (post-QC)	3505	9588	9588
Imputed variants shared between studies	2945	2945	2945
Genotype concordance ^c^	99.0%		NA
Imputed concordance ^c^	97.9%		NA
Analysis			
Hybrid log-linear model of gene-folate interactions	Yes	NA	Yes
Region-based analysis for rare variants	No	Yes	Yes
De novo variant detection	No	Yes	Yes

^a^ DNA microarray; ^b^ Probe hybridization enrichment and sequencing; ^c^ Discovery microarray vs. replication sequencing.

**Table 2:** Maternal demographics of discovery- and replication-phase samples used for DNA sequencing.

		CTD (*n* = 436)	Controls (*n* = 40) ^a^	*p*-Value ^b^
**Age at delivery (years)**		28.3 (5.79)	27.1 (5.71)	0.23
**Race**	Caucasian	298 (68.7%)	33 (86.8%)	0.05
	Hispanic	75 (17.3%)	1 (2.6%)	
	African American	29 (6.7%)	2 (5.3%)	
	Other	32 (7.3%)	2 (5.3%)	
**Education**	Less than 12 years	45 (10.3%)	1 (2.6%)	0.25
	High school or equivalent	100 (23.0%)	13 (34.2%)	
	1–3 years of college	133 (30.6%)	10 (26.3%)	
	Bachelors or 4 years college	156 (35.9%)	14 (36.8%)	
**Household income**	Less than $10,000	46 (24.1%)	3 (15%)	0.58
	$10,000–$30,000	57 (29.8%)	9 (45%)	
	$30,000–$50,000	39 (20.4%)	4 (20%)	
	Over $50,000	49 (25.7%)	4 (20%)	
**Folic acid supplementation**	Unsupplemented	176 (40.6%)	11 (28.9%)	0.17
	Supplemented	258 (59.4%)	27 (71.1%)	
**Alcohol consumption**	Unexposed	285 (65.7%)	24 (63.2%)	0.73
	Exposed	149 (34.3%)	14 (36.8%)	
**Cigarette smoking**	Unexposed	364 (84.1%)	30 (78.9%)	0.49
	Exposed	69 (15.9%)	8 (28.1%)	
	Missing	1	0	
**Maternal BMI**	Underweight (BMI < 18.5)	19 (4.5%)	2 (5.3%)	0.04
	Normal wt. (BMI 18.5–25)	190 (44.8%)	25 (65.8%)	
	Overweight (BMI 25–30)	116 (27.4%)	8 (21.1%)	
	Obese (BMI > 30)	99 (23.3%)	3 (7.9%)	
	Missing	10	0	

CTD, conotruncal defect; BMI, body mass index. ^a^ Demographic information was unavailable for 2 of the 40 control families included in this study. ^b^ Values based on Fisher’s exact test.

**Table 3:** Gene–folic acid interactions in the discovery and replication phases.

			*p*-Value			BFDP			Folate Supp.	RR and 95% CI		
Gene	SNP	Location	Disc.	Rep.	Meta	Disc.	Rep.	Meta		Discovery	Replication	Meta
SNPs with fetal gene-by-folate interactions												
*MTHFS*	rs12438477 ^a^	chr15:80,178,283	1.38 × 10^−3^	8.58 × 10^−3^	4.02 × 10^−5^	0.61	0.88	0.12	No	1.49 [1.12,1.97]	1.26 [0.81,1.95]	1.41 [1.11,1.79]
									Yes	0.83 [0.65,1.07]	0.59 [0.41,0.85]	0.74 [0.61,0.92]
*TCN2*	rs2301957 ^b^	chr22:31,018,817	1.2 × 10^−2^	1.04 × 10^−2^	4.66 × 10^−4^	0.83	0.88	0.39	No	1.14 [0.86,1.52]	1.55 [1.01,2.39]	1.25 [0.99,1.59]
									Yes	0.72 [0.56,0.92]	0.75 [0.52,1.08]	0.73 [0.59,0.89]
**SNPs with maternal gene-by-folate interactions**												
*NOS2*	rs2779248 ^c^	chr17:26,127,832	1.61 × 10^−3^	2.23 × 10^−2^	1.02 × 10^−4^	0.65	0.91	0.21	No	1.40 [1.05,1.87]	1.18 [0.76,1.82]	1.33 [1.04,1.69]
									Yes	0.77 [0.60,0.99]	0.59 [0.40,0.89]	0.72 [0.58,0.89]
*MTHFS*	rs7163338 ^d^	chr15:80,198,408	1.57 × 10^−2^	6.15 × 10^−3^	3.97 × 10^−4^	0.85	0.85	0.34	No	1.27 [0.96,1.67]	1.49 [1.02,2.17]	1.34 [1.07,1.67]
									Yes	0.82 [0.65,1.05]	0.73 [0.52,1.03]	0.79 [0.65,0.96]

Disc., discovery phase; Rep., replication phase; Meta, Meta-analysis; RR, relative risk; CI, Confidence Interval; BFDP, Bayesian Measure of the Probability; Folate supp., maternal periconceptional folic acid supplementation. ^a^ An additional 12 variants were identified within the same LD block with rs12438477 (i.e., Chr. 15, BP 80,157,583–80,179,847). ^b^ An additional 19 variants were identified within the same LD block with rs2301957 (i.e., Chr. 22, BP 31,004,127–31,024,856). ^c^ An additional 11 variants were identified within the same LD block with rs2779248 (i.e., Chr. 17, BP 26,103,034–26,129,996). ^d^ An additional 8 variants were identified within the same LD block with rs7163338 (i.e., Chr. 15, BP 80,166,597–80,198,417).

**Table 4:** Putative de novo mutations identified in the replication phase with target sequencing.

Variant	Gene	Region	MAF ^a^	Affected Regulatory Features ^b^	Predicted Effects
chr2:25460216C > T	*DNMT3A*	Intron	6.6 × 10^−6^	None	Enhances cryptic SSA
chr2:25532226T > C	*DNMT3A*	Intron	3.1 × 10^−4^	TFBS (YY1), DNase peak	
chr2:25548310C > T	*DNMT3A*	Intron	1.5 × 10^−4^	TFBS (GATA2, REST, ZNF263), DNase peak	Enhances cryptic SSD
chr2:25549757C > G	*DNMT3A*	Intron	None	TFBS (SETDB1), TF motif (EBF and EBF1), DNase Footprint, DNase peak	
chr2:25550547A > C	*DNMT3A*	Intron	None	TFBS (Freac-4)	
chr5:7875826A > G	*MTRR*	Intron	4.5 × 10^−4^	TFBS (Bhlhb2)	
chr12:16503384C > A	*MGST1*	Intron	None	TFBS (SMARCA4)	
chr15:80162657C > T	*MTHFS*	Intron	3.2 × 10^−3^	None	Enhances cryptic SSD
chr17:26101117A > G	*NOS2*	Intron	None	TFBS (NRSE, NRSF, NFAT5)	
chr17:26110823G > A	*NOS2*	Intron	2.0 × 10^−3^	TFBS (ESR1)	
chr18:671738C > T	*ENOSF1- TYMS*	Exon- Intron	6.2 × 10^−4^	TFBS (GATA1, POLR2A)	May provide new Poly-A signal for ENOSF1 3’UTR
chr22:31009950C > T	*TCN2*	Intron	None	DNase peak	Enhances cryptic SSA

^a^ MAF, minor allele frequency provided by the Genome Aggregation Database (gnomAD v3.1.2 using liftover from GRCh37 to GRCh38); ^b^ Regulatory features from RegulomeDB; TFBS, transcription factor binding site; SSA, splice site acceptor; SSD, splice site donor.

**Table 5:** Region-based analysis for rare variants.

Gene	Genomic Range	FB-SKAT	RV-TDT Burden Test
*DNMT3A*	chr2:25,454,844–25,566,444	0.628	0.755
*MRHFD2*	chr2:74,424,787–74,443,469	0.436	0.833
*MTRR*	chr5:7,868,246–7,902,214	0.727	0.867
*GLRX*	chr5:95,148,542–95,159,615	0.0147	0.392
*GCLC*	chr6:53,361,276–53,411,009	0.274	0.228
*SOD2*	chr6:160,099,857–160,115,421	0.217	0.434
*MGST1*	chr12:16,499,138–16,517,690	0.00298	0.67
*MTHFS*	chr15:80,134,820–80,190,536	0.24	0.605
*SHMT1*	chr17:18,230,107–18,267,686	0.066	0.212
*NOS2*	chr17:26,082,704–26,128,435	0.942	0.884
*TYMS*	chr18:657,140–674,589	0.238	0.322
*GPX4*	chr19:1,103,255–1,107,846	0.308	0.0384
*TCN2*	chr22:31,001,992–31,024,193	0.603	0.445

FB-SKAT, family-based sequence kernel association test; RV-TDT, rare-variant transmission disequilibrium test.

## Data Availability

Genetic data for individual participants is unavailable due to privacy restrictions. Please contact the corresponding author for additional information.

## Supplementary Materials

The following supporting information can be downloaded at: https://www.mdpi.com/article/10.3390/genes14010180/s1, Table S1: Number of sequenced cases and controls by infant-parental-enrollment status and study phase. Table S2: Maternal demographics by study phase. A. Demographics of sequenced participants in the discovery sample set. B. Demographics of the replication sample set. Table S3: Distribution of cardiac phenotypes by reported folate supplementation during the early prenatal period. Table S4: Extended list of gene–folic acid interactions. A. Fetal gene-by-folate interactions in the discovery and replication phases. B. Maternal gene-by-folate interactions in the discovery and replication phases. Figure S1: Linkage disequilibrium plots for variants with significant gene–folic acid interactions. Panel A. LD for variants within MTHFS showing potential fetal gene–folic acid interactions in the discovery phase (above) and the replication phase (bottom). Panel B. LD for variants within TCN2 showing potential gene–folic acid interactions in the discovery phase (above) and the replication phase (bottom). Panel C. LD for variants within NOS2 showing potential fetal gene–folic acid interactions in the discovery phase (above) and the replication phase (bottom). Panel D. LD for variants within MTHFS showing potential fetal gene–folic acid interactions in the discovery phase (above) and the replication phase (bottom). Figure S2: Workflow for gene–folic acid interaction analysis. File S1: Supplementary Materials for Statistical Methods.
