# Supplementary material for: Gene–Folic Acid Interactions and Risk of Conotruncal Heart Defects: Results from the National Birth Defects Prevention Study
Source: Genes (Basel). 2023 Jan 9;14(1):180. doi: 10.3390/genes14010180 (PMC9859210; doi:10.3390/genes14010180)
Supplement: Supplementary file 1 [file genes-14-00180-s001.zip › Figure S1.pdf]

Gene-folic acid interactions and risk of conotruncal heart defects: results from the National Birth Defects Prevention Study Daniel M. Webber, Ming Li, Stewart L. MacLeod, Xinyu Tang, Joseph W. Levy, Mohammad A. Karim, Stephen W. Erickson, Charlotte A. Hobbs*, and the National Birth Defects Prevention Study *Corresponding author: Charlotte A. Hobbs, E-mail: chobbs@rchsd.org,phone: 858-966-8852

Supplementary Figure S1. Linkage disequilibrium plots for variants with significant gene-folic acid interactions

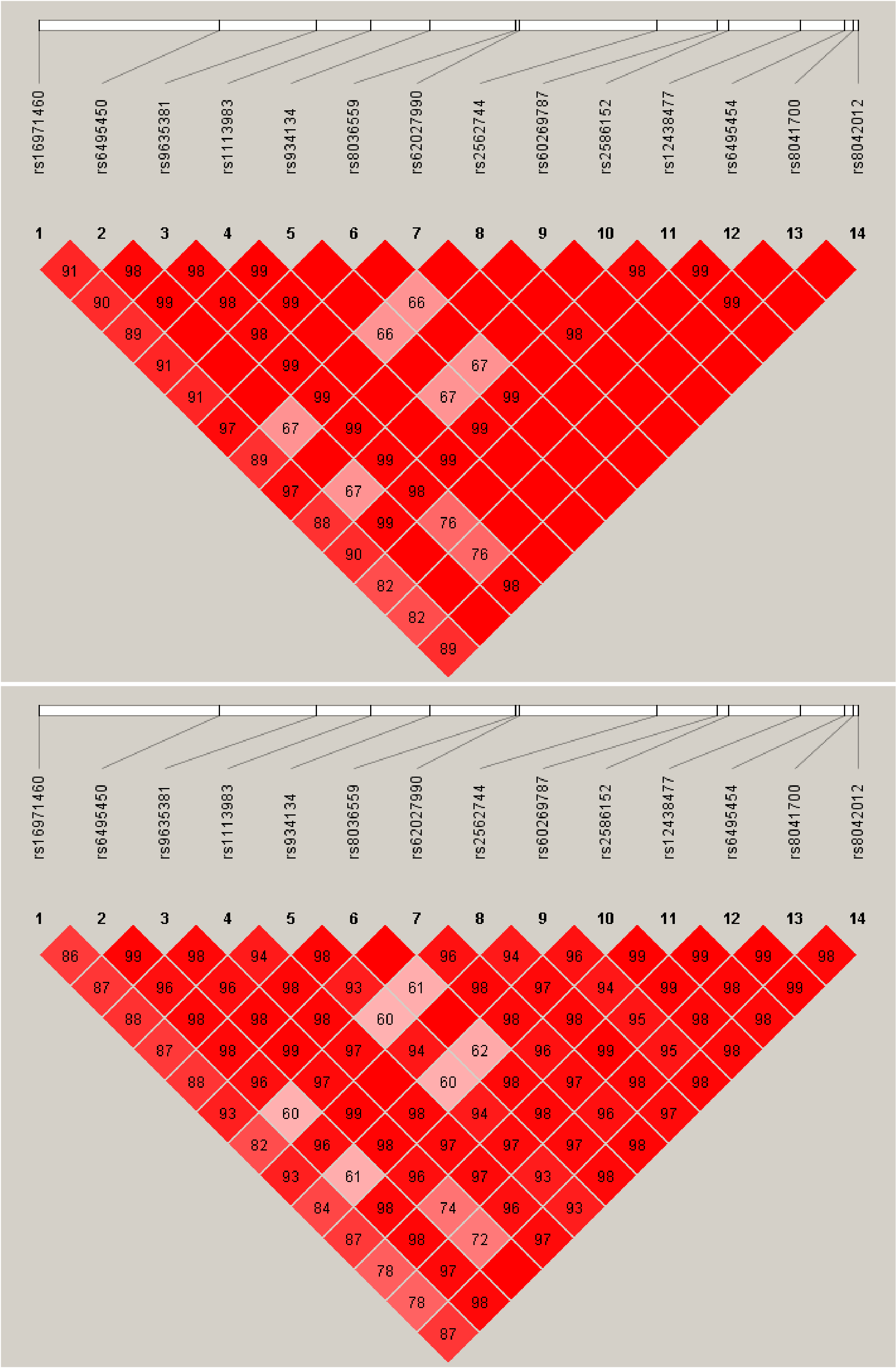

Supplementary Figure S1A. Linkage disequilibrium of variants with fetal gene-by-folate interactions in *MTHFS* from the discovery phase (top) and the replication phase (bottom).

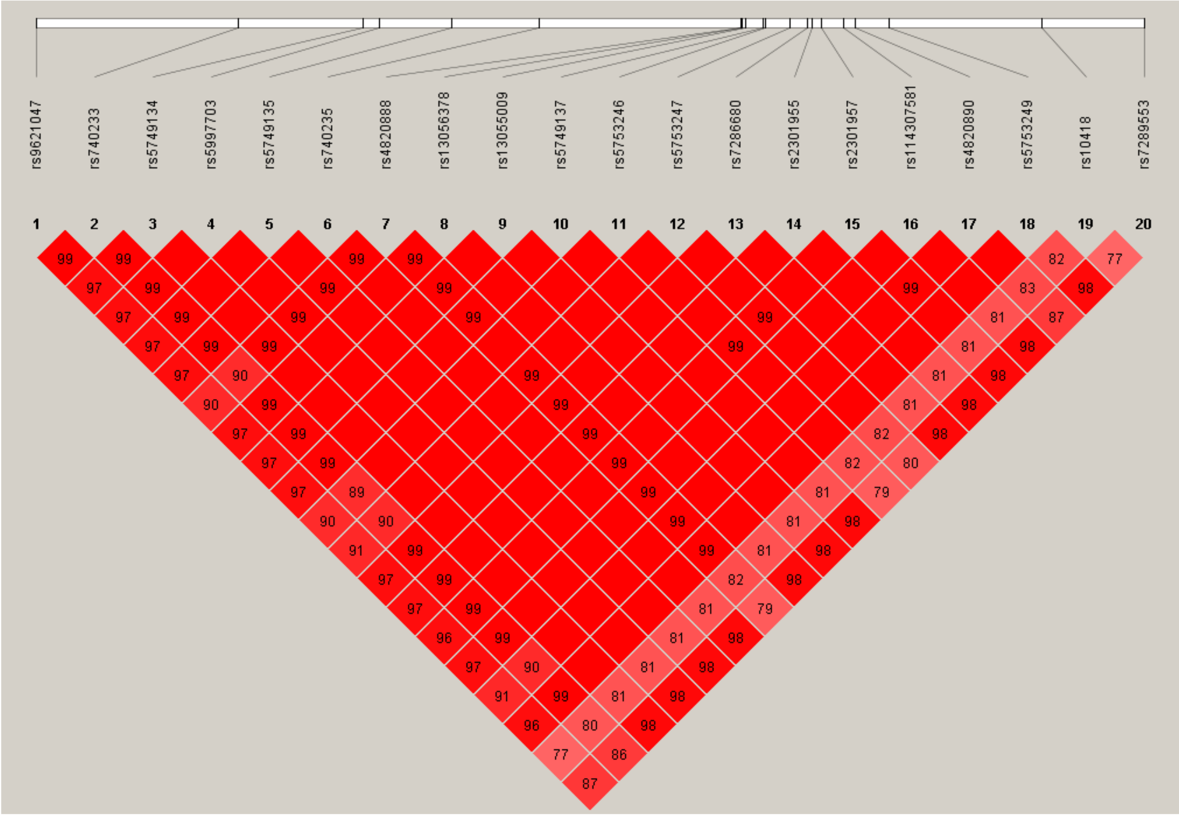

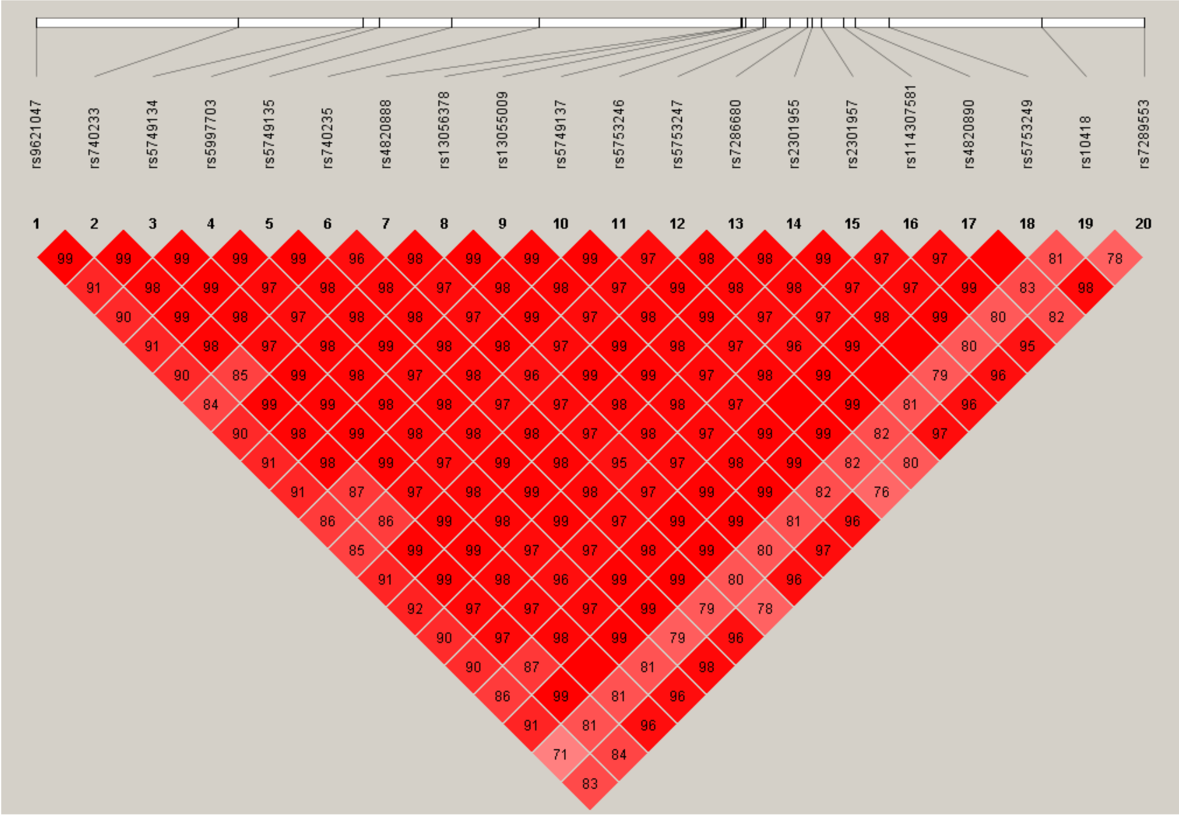

Supplementary Figure S1B. Linkage disequilibrium of variants with fetal gene-by-folate interactions in *TCN2* from the discovery phase (top) and the replication phase (bottom).

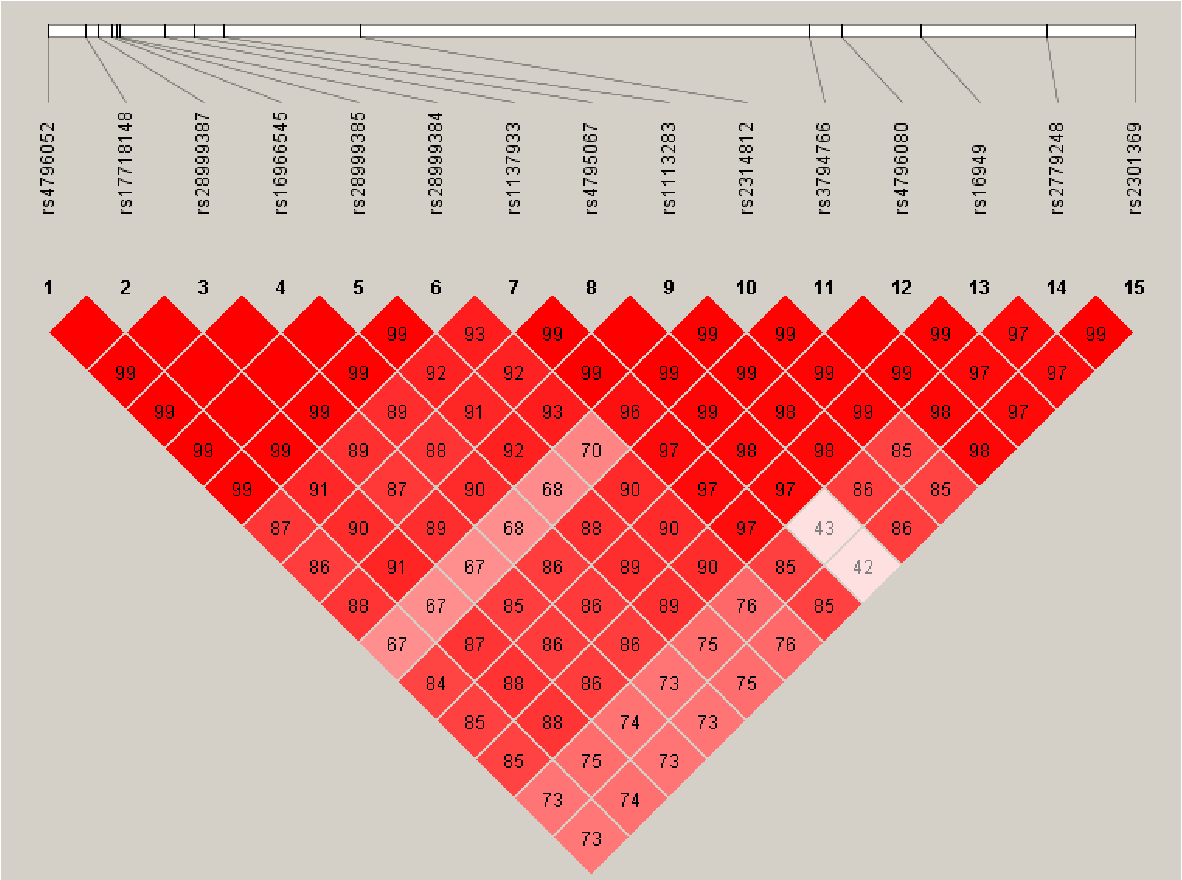

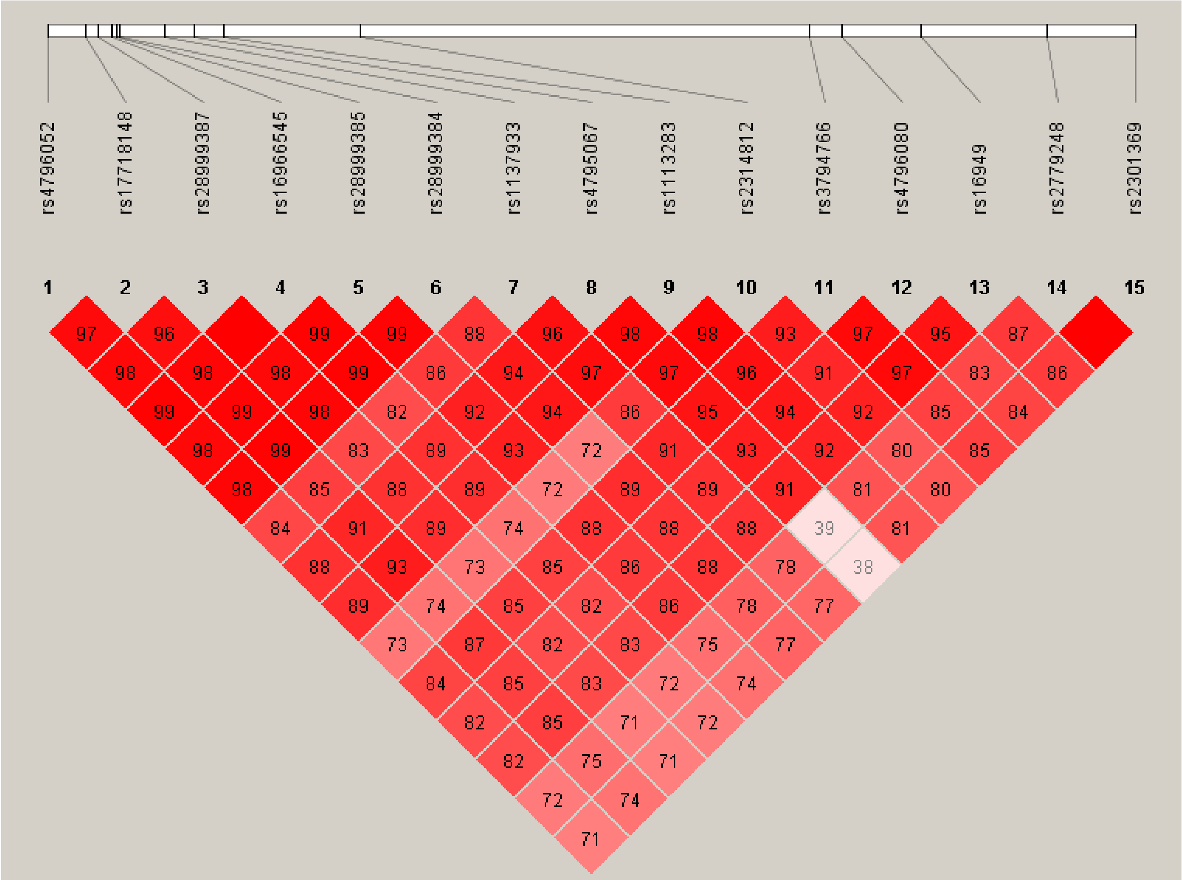

Supplementary Figure S1C. Linkage disequilibrium of variants with maternal gene-by-folate interactions in *NOS2* from the discovery phase (top) and the replication phase (bottom).

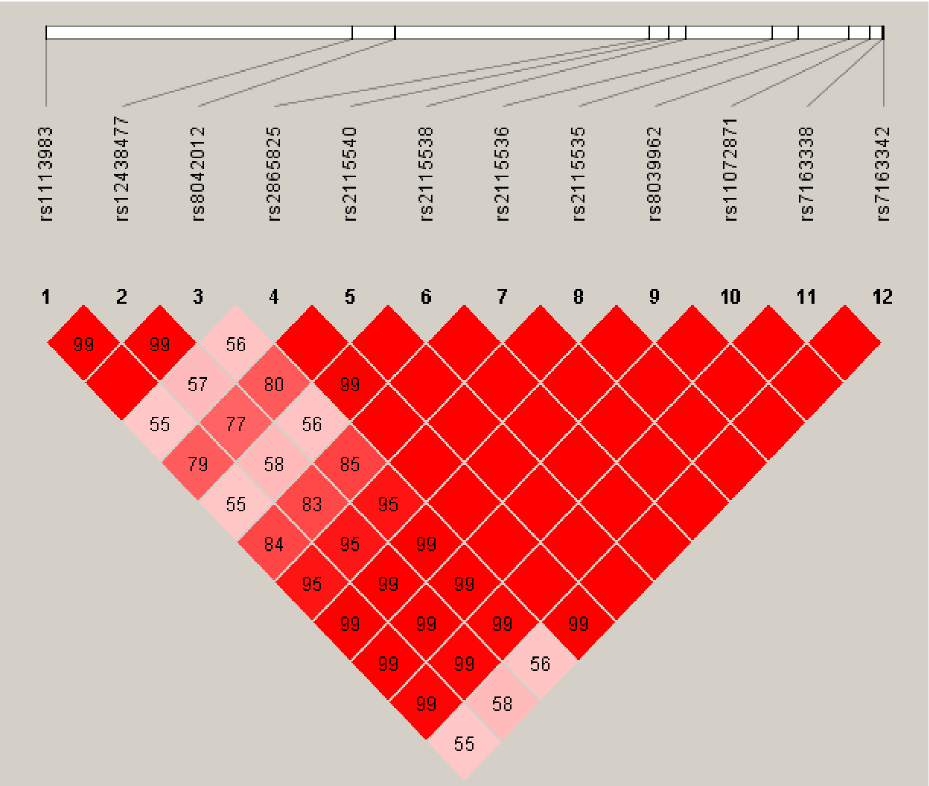

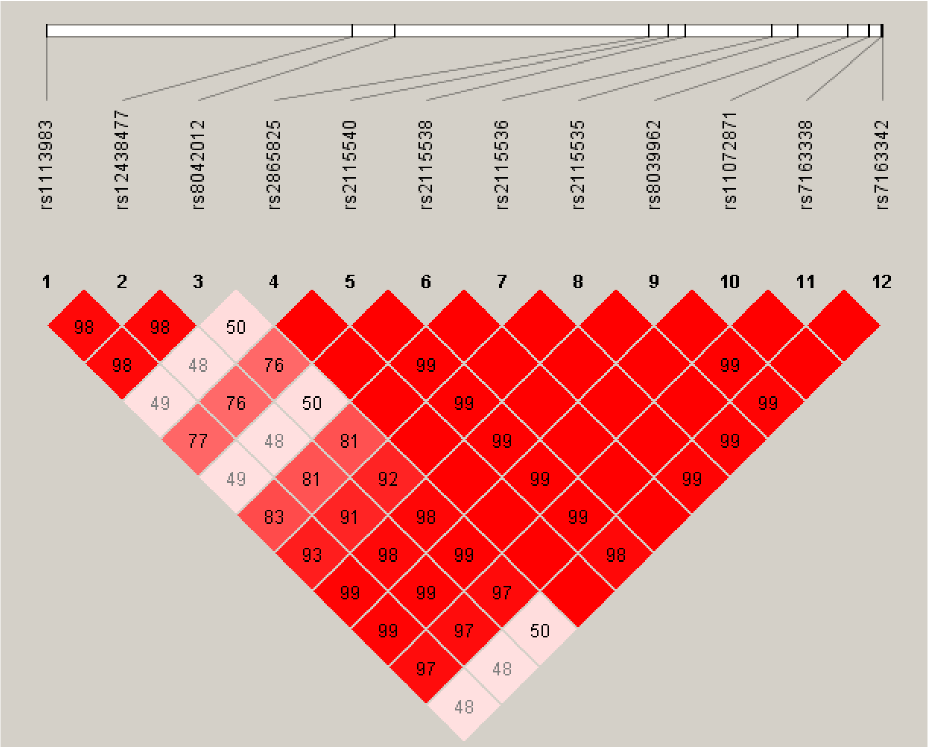

Supplementary Figure S1D. Linkage disequilibrium of variants with maternal gene-by-folate interactions in MTHFS from the discovery phase (top) and the replication phase (bottom).
