# Supplementary material for: Gene–Folic Acid Interactions and Risk of Conotruncal Heart Defects: Results from the National Birth Defects Prevention Study
Source: Genes (Basel). 2023 Jan 9;14(1):180. doi: 10.3390/genes14010180 (PMC9859210; doi:10.3390/genes14010180)
Supplement: Supplementary file 1 [file genes-14-00180-s001.zip › Supplementary_methods.pdf]

Supplementary Statistical Methods

In the following, we provide descriptions of the log-linear method proposed by Weinberg *et al*. and its extension used for gene-folate interaction analysis.

Log-linear method for case-parental-triad. Briefly, the log-linear method is initially proposed to estimate the relative genetic risk due to maternal and fetal genotypes using case-parent-triad data [1]. As discussed in Weinberg *et al.*, a mother-father-child (M, F, C) may have 15 possible combined genotypes. Without any genetic risk alleles, the theoretical population frequencies of case-parent triads with these genotypes can be modelled with 6 parameters for parental mating types in the following table (also see Table 1 in Weinberg *et al.*,).

Supplementary methods table 1. Frequencies of Case-parent-triad with and without Hardy-Weinberg Equilibrium (HWE)

	**Theoretical frequencies**	
**M,F,C Genotype**	**HWE assumption**	**Modeling without HWE**
Mating type 1:		
2,2,2	p^4	μ_1
Mating type 2:		
2,1,2	$p^3(1-p)$	μ_1
2,1,1	$p^3(1-p)$	μ_2
1,2,2	$p^3(1-p)$	μ_2
1,2,1	$p^3(1-p)$	μ_2
Mating type 3:		
2,0,1	$p^2(1-p)^2$	μ_3
0,2,1	$p^2(1-p)^2$	μ_3
Mating type 4:		
1,1,2	$p^2(1-p)^2$	μ_4
1,1,1	$2p^2(1-p)^2$	$2\mu_4$
1,1,0	$p^2(1-p)^2$	μ_4
Mating type 5:		
1,0,1	$p(1-p)^3$	μ_5
1,0,0	$p(1-p)^3$	μ_5
0,1,1	$p(1-p)^3$	μ_5
0,1,0	$p(1-p)^3$	μ_5
Mating type 6:		
0,0,0	$(1-p)^4$	μ_6

Assuming multiplicative (i.e., log-additive) risk per allele, the expected count in each cell (M,F,C) of the 15-nomial can be modeled in a log-linear form as

$$\ln\left[E(n_{M,F,C})\right] = \ln(\mu_j) + \beta_1 I_M + \beta_2 I_C + \ln(off) \qquad (1)$$

where $n_{M,F,C}$ is the expected cell counts for (M,F,C), μ_j, $j = 1,2,\ldots,6$ correspond to the six possible parental mating type categories in **Supplementary methods table 1**, I_M and I_C represents the number of copies of the variant allele (0, 1, or 2) carried by a mother and child, respectively. It is worthwhile to note that the log-linear model estimates the relative risk of maternal and fetal genotypes (*i.e.*, β_1 and β_2). It does not require samples from the control

families and can be regarded as a generalization of the transmission disequilibrium test (TDT). The theoretical details of the method are detailed elsewhere [1].

Log-linear method for hybrid design. Weinberg and Umback further extended the log-linear method to accommodate hybrid design with both case families and control families [2]. Following similar notations, the following model can be used assuming multiplicative (*i.e.*, log-additive) risk per allele:

$$\ln\left[E\left(n_{M,F,C,D}\right)\right] = \ln\left(\mu_j\right) + \gamma I_{(D=1)} + \beta_1 I_{(D=1)} I_M + \beta_2 I_{(D=1)} I_C + \ln(off) \qquad (2)$$

where μ_j, $j = 1,2,\ldots,6$, I_M and I_C have the same definition as for case-parental-triad; $I_{(D=1)}$ is the indicator variable for case families ($I_{(D=1)} = 1$) or control families ($I_{(D=1)} = 0$).

Log-linear method for gene-by-environment interaction. We and others have previously used an extended log-linear model to evaluate gene-by-folate interaction associated with the risk of congenital heart defects [3]. The following model was fitted:

$$\ln\left[E\left(n_{M,F,C,D,E}\right)\right] = \ln\left(\mu_j\right) + \delta I_{(E=1)} + \gamma I_{(D=1)} + \beta_1 I_{(D=1)} I_M + \beta_2 I_{(D=1)} I_C$$
$$+\beta_3 I_{(D=1)} I_{(E=1)} I_M + \beta_4 I_{(D=1)} I_{(E=1)} I_C + \ln(off) \qquad (3)$$

Based on this extended model, the maternal and fetal gene-by-folate interactions can be evaluated by using a Wald test for parameters β_3 and β_4. The relative risk for maternal effect among families unexposed to folate can be estimated by $\exp(\beta_1)$, and that among families exposed to folate can be estimated by $\exp(\beta_1 + \beta_3)$. The relative risk for fetal effects among exposed and unexposed families can be estimated in a similar fashion. As we discussed in the description of the log-linear model for case-parental-triads, the relative risks of maternal and fetal genotypes are not only based on the comparison between cases and controls and can be estimated without control families through a reduced model.

Meta-Analysis of the discovery and replication phases. We used software *Metal* to conduct fixed effect meta-analysis integrating the estimates from the discovery and replication phases [4]. Inverse variance weighting was used in the meta-analysis. Briefly, let β_d (se_d) and β_r (se_r) be the effect size (standard error) estimates in the discovery and replication phases, respectively. The overall effect and standard error can be estimated as:

$$\beta_{overall} = (\beta_d/{se_d}^2 + \beta_r/{se_r}^2)/(1/{se_d}^2 + 2/{se_d}^2);\ se_{overall} = \sqrt{{se_d}^2 + {se_r}^2}$$

Bayesian false discovery probability (BFDP). We used BFDP to assess the noteworthiness of an association by balancing the costs of false discovery and non-discoverye [5]. The method is available in R package *gap.* Following the notations in *Wakefield J*, BFDP reflects the probability of being null hypothesis given an estimate of the log relative risk ($\hat{\theta}$), the variance of this estimate (V), the prior variance (W), and the prior probability of a non-null association (π_0). Denote $r = \frac{W}{V+W}$ and $Z = \frac{\hat{\theta}}{\sqrt{V}}$. An appropriate Bayes factor (ABF) and prior odds (PO) can be obtained as

$$ABF = \frac{p(\hat{\theta}|H_0)}{p(\hat{\theta}|H_1)} = \sqrt{\frac{V+W}{V}} \exp\left[-\frac{\hat{\theta}^2}{2} \times \frac{W}{V(V+W)}\right] = \frac{1}{\sqrt{1-r}} \exp\,[-\frac{Z^2}{2} r]$$

$$PO = \frac{\pi_0}{1-\pi_0}$$

The BFDP can then be estimated as:

$$BFDP = \frac{ABF \times PO}{ABF \times PO + 1}$$

Bibliography

1. Weinberg CR, Wilcox AJ, Lie RT. A log-linear approach to case-parent-triad data: assessing effects of disease genes that act either directly or through maternal effects and that may be subject to parental imprinting. Am J Hum Genet. 1998;62(4):969-78. doi: 10.1086/301802. PubMed PMID: 9529360; PMCID: PMC1377041.
2. Weinberg CR, Umbach DM. A hybrid design for studying genetic influences on risk of diseases with onset early in life. Am J Hum Genet. 2005;77(4):627-36. Epub 20050831. doi: 10.1086/496900. PubMed PMID: 16175508; PMCID: PMC1275611.
3. Hobbs CA, Cleves MA, Macleod SL, Erickson SW, Tang X, Li J, Li M, Nick T, Malik S, National Birth Defects Prevention S. Conotruncal heart defects and common variants in maternal and fetal genes in folate, homocysteine, and transsulfuration pathways. Birth Defects Res A Clin Mol Teratol. 2014;100(2):116-26. Epub 20140218. doi: 10.1002/bdra.23225. PubMed PMID: 24535845; PMCID: PMC4118819.
4. Willer CJ, Li Y, Abecasis GR. METAL: fast and efficient meta-analysis of genomewide association scans. Bioinformatics. 2010;26(17):2190-1. Epub 20100708. doi: 10.1093/bioinformatics/btq340. PubMed PMID: 20616382; PMCID: PMC2922887.
5. Wakefield J. A Bayesian measure of the probability of false discovery in genetic epidemiology studies. Am J Hum Genet. 2007;81(2):208-27. Epub 20070703. doi: 10.1086/519024. PubMed PMID: 17668372; PMCID: PMC1950810.
