# Supplementary figures and images for: Gene–Folic Acid Interactions and Risk of Conotruncal Heart Defects: Results from the National Birth Defects Prevention Study
Source: Genes (Basel). 2023 Jan 9;14(1):180. doi: 10.3390/genes14010180 (PMC9859210; doi:10.3390/genes14010180)

## Supplementary Figure S2. Workflow for gene-folic acid interaction analysis

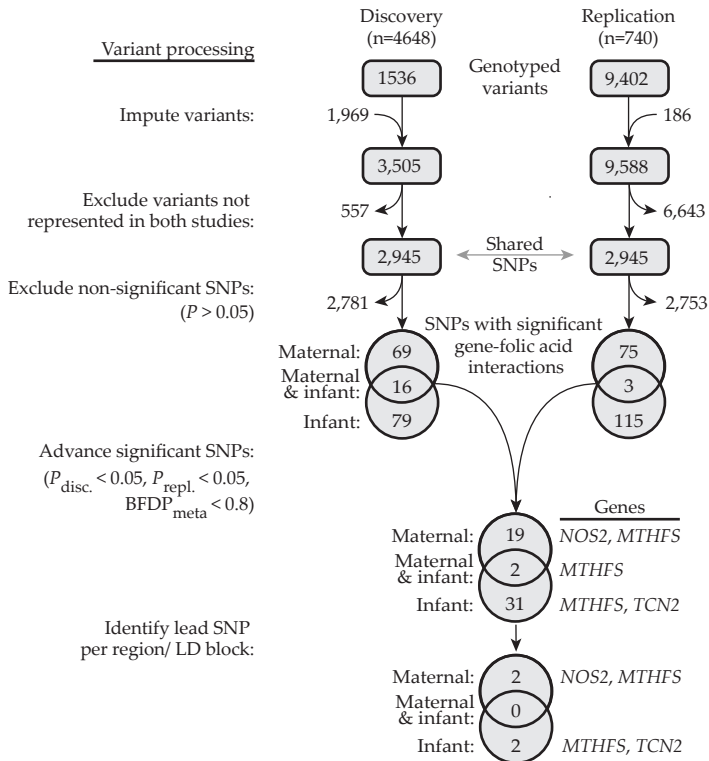

Supplement: Supplementary file 1 [file genes-14-00180-s001.zip › Figure S2.pdf]
